# Titanium Culture Vessel Presenting Temperature Gradation for the Thermotolerance Estimation of Cells

**DOI:** 10.34133/cbsystems.0049

**Published:** 2023-08-07

**Authors:** Chikahiro Imashiro, Yangyan Jin, Motoaki Hayama, Takahiro G. Yamada, Akira Funahashi, Katsuhisa Sakaguchi, Shinjiro Umezu, Jun Komotori

**Affiliations:** ^1^ Graduate School of Engineering, The University of Tokyo, Tokyo 113-0033, Japan.; ^2^ Department of Mechanical Engineering, Keio University, Yokohama, Kanagawa 223-0061, Japan.; ^3^ School of Integrated Design Engineering, Graduate School of Science and Technology, Keio University, Yokohama, Kanagawa 223-0061, Japan.; ^4^ Department of Biosciences and Informatics, Keio University, Yokohama, Kanagawa 223-0061, Japan.; ^5^ Department of Integrative Bioscience and Biomedical Engineering, Graduate School of Advanced Science and Engineering, Waseda University, TWIns, Tokyo 162-8480, Japan.; ^6^ Department of Modern Mechanical Engineering, Waseda University, Tokyo 169-8555, Japan.

## Abstract

Hyperthermia can be induced to exploit the thermal intolerance of cancer cells, which is worse than that of normal cells, as a potential noninvasive cancer treatment. To develop an effective hyperthermia treatment, thermal cytotoxicity of cells should be comprehensively investigated. However, to conduct such investigations, the culture temperature must be accurately regulated. We previously reported a culture system in which the culture temperature could be accurately regulated by employing metallic culture vessels. However, appropriate temperature conditions for hyperthermia depend on the cell species. Consequently, several experiments need to be conducted, which is a bottleneck of inducing hyperthermia. Hence, we developed a cell culture system with temperature gradation on a metallic culture surface. Michigan Cancer Foundation-7 cells and normal human dermal fibroblasts were used as cancer and normal cell models, respectively. Normal cells showed stronger thermal tolerance; this was because the novel system immediately exhibited a temperature gradation. Thus, the developed culture system can be used to investigate the optimum thermal conditions for effective hyperthermia treatment. Furthermore, as the reactions of cultured cells can be effectively assessed with the present results, further research involving the thermal stimulation of cells is possible.

## Introduction

Despite the development of new therapies, cancer remains the leading cause of death [1]. Surgery, chemotherapy, and radiation therapy are the main cancer treatments worldwide, although each strategy has its own disadvantages, such as side effects and invasiveness [2–4]. Hyperthermia has been studied as a potential cancer therapy owing to its minimal side effects and invasiveness [5]. Accordingly, in previous reports, ultrasound, magnetic field, or laser for clinical treatment have been utilized for administrating temperature [6–11]. Cancer cells are more vulnerable to thermal stimulation compared to normal cells, which can be exploited via hyperthermia by exposing the diseased area to a certain thermal dose [12]. Cancer cells are selectively killed because thermal cytotoxicity of cancer cells is more prominent than that of normal cells with respect to the same thermal stimulation. Furthermore, hyperthermic treatment can be combined with other therapies, such as chemotherapy, because chemical reactions can be affected by thermal stimulation as well [13]. However, to develop this treatment, the thermal cytotoxicity of cells should be strictly investigated.

In several studies, cultured cells have been exposed to thermal stimulation by regulating the cell culture incubator temperature [14–17]. However, in such experiments, the thermal stimulation of cells cannot be immediately and accurately regulated because the temperature the cells are exposed to is indirectly regulated via the incubator atmosphere. Thus, we fabricated a culture system in which the temperature to which cells were exposed could be accurately controlled using a metallic culture vessel and Peltier element to construct a database with precise information on the thermotolerance of each cell type [18,19]. The advantage of a metallic culture vessel, owing to its capability of accurate temperature regulation, has been demonstrated experimentally, resulting in an effective culture system for investigating the thermal tolerance of cells. However, appropriate temperature conditions for hyperthermia depend on the cell species. Consequently, several experiments are required, which represents a bottleneck in the development of hyperthermia therapy. Thus, there is an urgent need for a novel and effective method to measure thermal cytotoxicity within a certain range of culture temperatures.

**Fig. 1.**
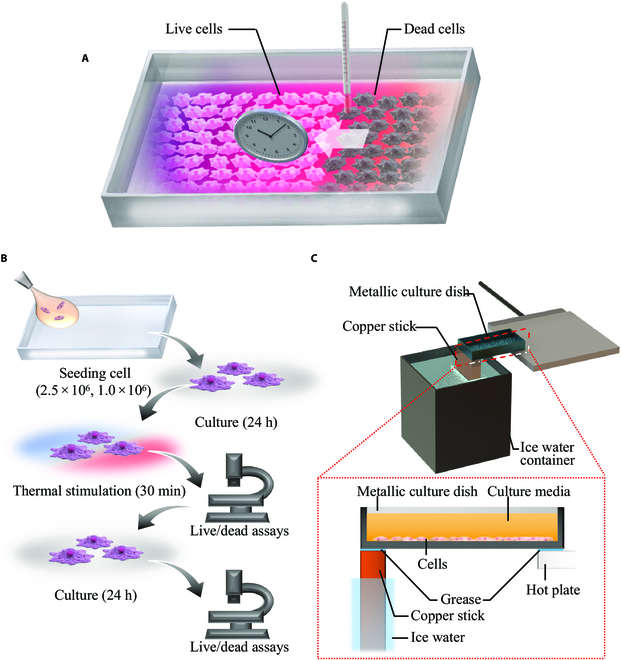
Schematic of the research and developed system. (A) The culture system concept. Owing to temperature gradation, the thermal responses of the cells to comprehensive temperature conditions can be observed simultaneously. Furthermore, after thermal stimulation, cell death is observed. (B) Experimental procedure used in this study. (C) Schematic of the culture system in which temperature gradation is presented. The metallic culture vessel is placed on a heating plate and copper stick soaked in ice water.

Therefore, in this study, we developed a cell culture system with temperature gradation on a metallic culture surface, as shown in Fig. 1A. This temperature gradation facilitates the simultaneous evaluation of cellular reactions over a wide range of temperature stimuli. In addition, because of the metallic culture surface, the temperature stimulation of the cells can be immediately and accurately controlled. Thereafter, Michigan Cancer Foundation-7 (MCF-7) cells and normal human dermal fibroblasts (NHDFs), which serve as models of cancer and normal cells, respectively, were cultured with the proposed device and exposed to thermal stimulation to investigate the reaction of each cell type to thermal stimulus (see Fig. 1B). The results characterize the candidate critical condition for hyperthermia, which is useful for improving hyperthermia treatment. In addition, different cell reactions to thermal stimuli were observed at different seeding densities. Thus, the proposed device developed to investigate the effective thermal conditions for hyperthermia is expected to aid in the development of noninvasive cancer treatments.

## Methods

### Modification and evaluation of the culture surface

Fine-particle peening (FPP) is a surface treatment method in which fine media (<200 μm in diameter) are projected onto a material surface at high speed. Roughness is generated on the material surface by plastic deformation owing to particle collisions. FPP for surface modification was performed under the conditions listed in Table. To evaluate the culture surface, arithmetic mean roughness, *Sa*, and maximum height roughness, *Sz*, values were measured on the obtained surface profile using a laser microscope (VK-X1000, KEYENCE Corp., Osaka, Japan).

**Table.** Conditions of FPP.

Particle	Aluminum oxide (Al_2_O_3_)
Particle diameter	#60 (median particle diameter 212–300 μm)
	#180 (median particle diameter 53–90 μm)
Projection method	Air blasting
Air pressure	0.6 MPa

### Cell culture

MCF-7 (RIKEN BRC, Saitama, Japan) and NHDF cells (Cosmo Bio Co., Ltd., Tokyo, Japan) were cultured in Dulbecco’s modified Eagle’s medium (11965092; Thermo Fisher Scientific Inc.) supplemented with 10% fetal bovine serum (Funakoshi Co., Ltd., Tokyo, Japan) and 1% penicillin (15140122; Thermo Fisher Scientific Inc.) at 37 °C under 5% CO_2_. Cells were detached using 0.05% trypsin-ethylenediaminetetraacetic acid (25300; Life Technologies, Carlsbad, CA, USA). Except for the experiments, the cells were cultured in conventional plastic culture dishes.

**Fig. 2.**
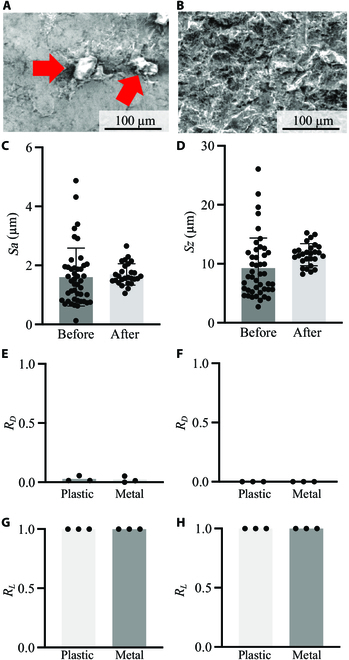
Fabrication and cell adaptability of the metallic culture surface. SEM images before (A) and after (B) FPP. Red arrows indicate partial bumps or dips. Comparisons between arithmetic mean roughness, *Sa*, (C) and maximum height roughness, *Sz*, (D) values before and after the FPP. Ratios of detached MCF-7 (E) and NHDF (F) to the number of seeded cells after 24-h culture (*R_D_*, ratio of detached cells). Ratios of live cells area to all cell area *(R_L_*) after 24-h culture (G: MCF-7 and H: NHDF).

To demonstrate the cell adaptability (Fig. 2E to H), 1.0 × 10^6^ cells were seeded into the metallic culture vessels and cultured for 24 h, while cells with the same density on culture surfaces were seeded and cultured in 35-mm plastic culture dishes. After culturing, the supernatant was collected to count the number of cells detached from the culture surface. To measure cell viability, live and dead cells were stained with calcein-AM and propidium iodide, respectively. The ratios of live-cell areas were used to determine cell viability.

### Finite element method

The 3-dimensional (3D) model of the experimental equipment for the FEM analysis comprised the vessel, hot plate, copper stick, ice water, and its container and constructed using COMSOL Multiphysics 5.6a. The model shown in Fig. S4 was built at a scale of 1:1 using the fluid flow and heat transfer modules. The surrounding temperature was set to 37 °C, and boundary conditions were applied as shown in Fig. S4. The flow of the culture medium inside the vessel was modeled as an incompressible laminar flow.

### Image processing

Image processing for the quantification of thermal tolerance was performed as follows. The distribution of live and dead cells was quantified. The ratio of live cells to entire cell population was plotted, and moving average was applied with a period of 100 pixels to smoothen the error due to the random cell distribution. Here, 100 pixels corresponded to 0.171 mm, which corresponded to 0.141 °C.

### Cleaning the device

The culture vessels were cleaned using the following procedure. The device was first cleaned by soaking overnight in a diluted detergent (7X cleaning solution, Funakoshi Co., Ltd., Tokyo, Japan) and then ultrasonically cleaned with diluted detergent, ultrapure water, and 70% ethanol for 15 min each. An autoclave was used for sterilization.

### Statistical tests

All statistical tests to compare the temperature sensitivity to culture duration after thermal treatment and cell types were performed using 2-way analysis of variance (ANOVA) (significance level α = 0.05).

## Results

### Development of the culture system with temperature gradation

The developed system comprised a metallic culture vessel with an 1,800-mm^2^ culture surface area made from commercial-grade pure titanium and temperature regulation system, as shown in Fig. 1C. To realize temperature gradation, one side of the vessel was heated and the other side was cooled. A hot plate (PH200-100-PCC10A, AS ONE, Osaka, Japan) was used to heat the vessel. A copper stick soaked in ice water was used as the cooler. The temperature of iced water was maintained at 0 °C, which provided a stable and strong cooler. Furthermore, the contact surface between the vessel and copper stick/hot plate was covered with grease (PC007, Nissei Industries Co., Ltd., Shizuoka, Japan) for effective temperature transmission.

### Surface modification of the culture vessel

The metallic vessel was fabricated using a metallic 3D printer (EOSINT M 280, Electro-Optical Systems Inc., Phoenixville, PA, USA); thus, the morphology of the culture surface was not regulated, as shown in Fig. 2A and Fig. S1A. Because the morphology of the culture surface to which cells adhere affects their activities and functions [20,21], a homogeneous culture surface should be realized. Therefore, FPP was performed. Observe that the FPP-treated metallic culture surface exhibited good cell adhesion and biocompatibility as previously reported [22,23].

Consequently, a relatively homogeneous culture surface was obtained, as shown in Fig. 2B and Fig. S1B. Furthermore, to show homogeneity, the arithmetic mean roughness (*Sa*) and maximum height roughness (*Sz*) values of the culture surface with and without FPP were considered as qualitative indices and measured (Fig. 2C and D). This result indicates that the variability in culture surface roughness decreased owing to the FPP treatment. The definitions of *Sa* and *Sz* are provided in Note S1.

### The adaptability of cells to the metallic vessel

Cell adhesion and viability were measured to evaluate the adaptability of the cells to the metallic vessels, as shown in Fig. 2E to H. Both MCF-7 and NHDF showed good cell adhesion ratios and viability in the metallic vessel compared with the plastic vessel after 24 h of culturing. Although plastic materials generally require surface coating or modification, a metallic culture surface, including the considered titanium surface does not require such postprocessing, which is an advantage of metallic culture surfaces.

### Evaluation of the system

**Fig. 3.**
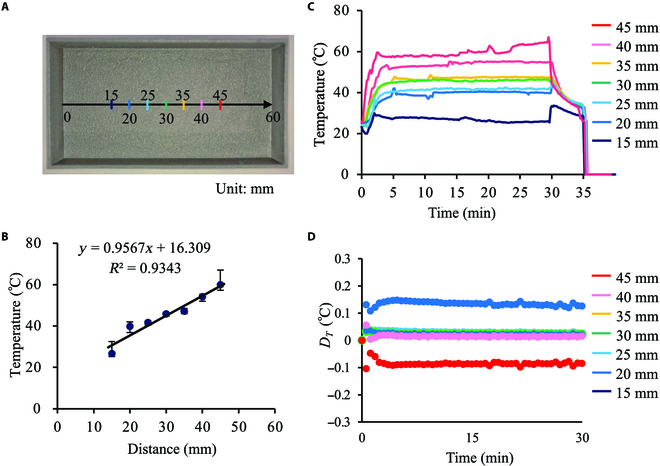
Evaluation of the culture vessel temperature. (A) Measurement positions on the culture surface. (B) Temperature gradation on the culture surface. The upper and lower bounds of bars denote the maximum and minimum values, respectively. (C) Temperature history of the culture surface. After the 30-min temperature control, the metallic vessel was removed from the system, which diminished the temperature gradient. (D) Temperature difference between cells and culture surface calculated by finite element analysis.

The temperatures of the device and cells exposed to heat were evaluated, as shown in Fig. 3. Figure 3A shows the positions at which the temperature was monitored on the culture surface. Figure 3B shows the temperature gradient on the culture surface. Each temperature obtained was averaged over 5 to 30 min after the start of temperature regulation. Furthermore, Fig. S2 presents the temperature measurement method. The temperature history was measured at each point, as shown in Fig. 3C. After 30 min of temperature control, the metallic vessel was removed manually from the system. As evident, the temperature of the vessel surface can be immediately regulated at each point. The temperature increase after 30 min near the hot plate is theoretically peculiar because the system loses contact with the heat source. However, we manually removed the vessels from the system having both the hot plate and cold copper stick, which may have caused a discrepancy in the timing of contact between the heat source and cooling source. Thus, a temperature increase occurred, which could be resolved through the mechanization of the experimental system. Furthermore, the temperature difference between the cells and culture vessel at each point was analyzed using finite element analysis (COMSOL Ver. 5.6; COMSOL AB, Stockholm, Sweden), as shown in Fig. 3D. The figure depicts the existence of very small discontinuities between the cell temperatures and culture surface, underlining the exposure of cultured cells to the thermal stimulation via the vessel.

**Fig. 4.**
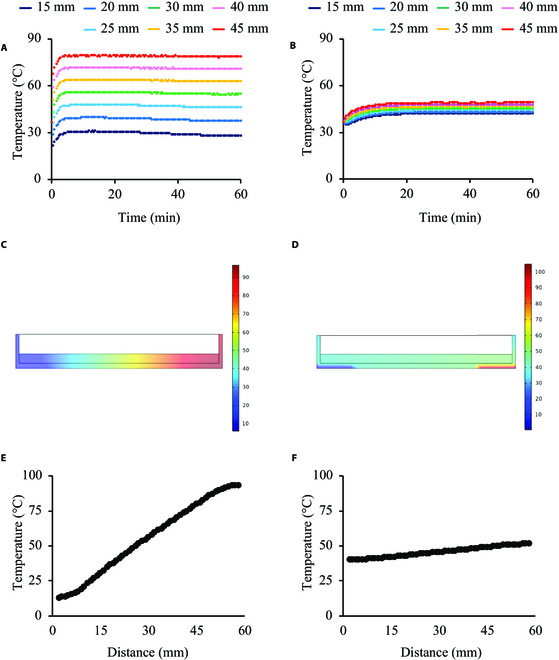
Comparison of the presented temperature distribution on metallic (A and C) and plastic (B and D) culture vessels using simulations. The temperature at each position was simulated every 30 s using metallic (A) and plastic (B) culture vessels. The corresponding positions are shown in Fig. 4. Cross-sectional images of the vessels made of metallic (C) and plastic (D) materials after 20 min of driving are shown, and quantitative results are also shown in (E) and (F), respectively. The color bars indicate temperature (°C).

The advantage of employing a metallic material as the culture vessel was also demonstrated by finite element analysis. The temperature gradation and history of the metallic and polystyrene vessels are compared in Fig. 4A and B. The metallic vessel exhibited a wide range of temperature gradations and immediate regulation. However, when polystyrene (conventionally employed) was used as the vessel material, a narrower temperature gradation and slower temperature regulation speed were observed. Although the polystyrene vessel achieved stable condition after 20 min of driving, the metallic vessel required less than 5 min. To illustrate the temperature gradation after reaching equilibrium, animations of the temperature variations are provided in supplementary movies. In Movies S1 and S2, polystyrene and titanium were used as the material of the vessels, respectively. The movies contained 241 frames simulated every 5 s. The final frame of each condition 20 min after starting the experiment is shown in Fig. 4C and D. Figure 4E and F shows the temperature gradients of the frames depicted in Fig. 4C and D in a quantitative manner. The homogeneous temperature distribution along the short direction is illustrated in Fig. S3.

Furthermore, the primary stimulus should be considered as the temperature factor in this system; however, the flow stimulus may be provided to cells via convection, which should also be considered. Thus, in this study, we evaluated the flow of the medium during temperature regulation via the application of microparticles (FA-207; Sinloihi Co., Ltd., Kanagawa, Japan), which resulted in no observed flow of the culture medium (data not shown). Thus, it can be argued that temperature is the only factor stimulating cellular activity in this system.

### Thermal tolerance of cells

**Fig. 5.**
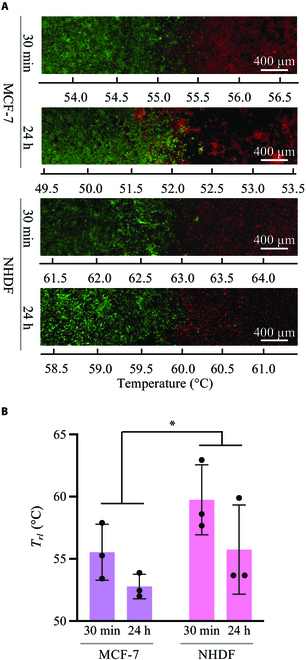
Effects of thermal stimulation on cells with a seed number of 2.5 × 10^6^. (A) Typical fluorescent images of MCF-7 and NHDF cultured for 30 min or 24 h after 30-min thermal stimulation. (B) Qualitative results for each condition (*n* = 3; mean ± SD; *P < 0.05). *T_rl_*, the temperature at which the ratio of the live cells reached a value of 0.5.

The system and temperature gradations shown in Fig. 3 were used to evaluate the thermal tolerance of the cells, which is illustrated in Fig. 1B. To compare the differences in thermal tolerance between different cell species, MCF-7 and NHDF were used in this study. A total of 2.5 × 10^6^ cells were seeded into the vessel with a culture surface area of 1,800 mm^2^ and 6-ml of culture media and cultured for 24 h. Subsequently, they were exposed to thermal stimulation for 30 min. Figure 5 shows the results of the experiment immediately after thermal stimulation and after 24 h of culturing following thermal stimulation. In Fig. 5A, typical images depicting cell viability are shown, while the quantitative evaluation is shown in Fig. 5B. Live and dead cells were stained with calcein-AM (Dojindo, Tokyo, Japan) and propidium iodide (169-26281, FUJIFILM Wako Pure Chemical Corporation, Osaka, Japan), respectively. Images were captured using an upright fluorescence microscope (LV100ND, Nikon Corporation, Tokyo, Japan). Green and red represent live and dead cells, respectively. As shown in Fig. 5A, the borders of live/dead cells were observed; accordingly, a gradation in the distribution of live/dead cells was discovered. To quantify these distributions, the areas of live and dead cells were measured using ImageJ (Ver 2.1.0/1.53c, National Institutes of Health, Bethesda, MD, USA), and the ratio of the surface area of live cells to that of all cells at the presented temperature was quantified. The temperature at which the ratio of live cells reached a value of 0.5, *T_rl_*, was quantitatively measured, as shown in Fig. 5B, which was called the threshold temperature. Furthermore, the substantial effects of the cell type difference between MCF-7 and NHDF on the threshold temperature were confirmed (2-way ANOVA, *P* = 0.0432 < 0.05), with the temperature threshold tending to be higher for NHDF than for MCF-7 cells. This demonstrates that NHDF exhibited a stronger thermal tolerance than MCF-7.

### Cell density exclusively affecting the thermal tolerance of NHDF

**Fig. 6.**
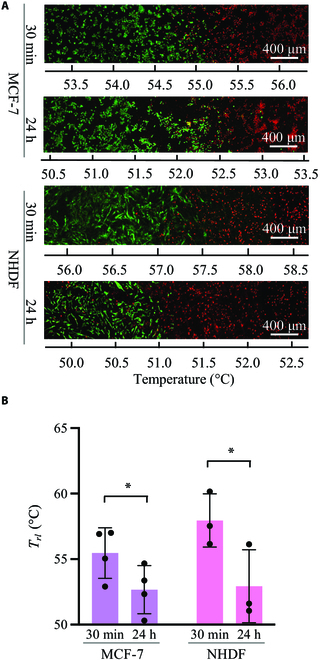
Effects of thermal stimulation on cells seeded at 1.0 × 10^6^. (A) Typical fluorescent images of MCF-7 and NHDF cultured for 30 min or 24 h after 30-min thermal stimulation. (B) Qualitative results for each condition (*n* = 4; mean ± SD; *P < 0.05). *T_rl_*, the temperature at which the ratio of the live cells reached a value of 0.5.

**Fig. 7.**
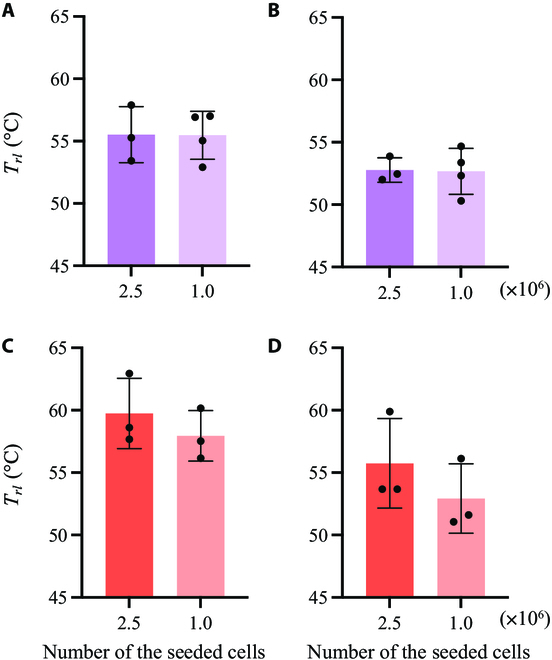
Comparison of the effects of cell density on thermotolerance. (A) MCF-7/30 min of culture after 30-min thermal stimulation. (B) MCF-7 cultured for 24 h after 30-min thermal stimulation. (C) NHDF cultured for 30 min after 30-min thermal stimulation. (D) NHDF cultured for 24 h after 30-min thermal stimulation. *T_rl_*, the temperature at which the ratio of the live cells reached a value of 0.5.

The thermal tolerances of cells with different cell densities were confirmed. The experiments following the procedure shown in Fig. 1B were performed with a smaller cell density of 1.0 × 10^6^ to 1,800 mm^2^ less than that considered in the experiment shown in Fig. 5. Figure 6A and B show typical fluorescence images and quantified results, respectively, which are similar to those shown in Fig. 5. Green and red represent live and dead cells, respectively. As shown in Fig. 6A, the borders of live/dead cells were observed under each condition. Instead of the effects of different cell types, which were identified at high cell densities (Fig. 5), the culturing time after thermal treatment significantly affected the threshold temperature at low cell densities (2-way ANOVA, *P* = 0.0067 < 0.05). To demonstrate the effect of cell density variation on thermal tolerance, the quantified results shown in Figs. 5B and 6B were compared from the perspective of cell density depicted in Fig. 7. Under our experimental conditions, the thermotolerance of NHDF was strengthened owing to the increased cell density. In contrast, MCF-7 cells, which were used as the cancer model, did not show this increase.

## Discussion

In this study, a novel method for measuring the thermal tolerance to a certain range of culturing temperatures was developed to obtain an effective hyperthermia treatment. Accordingly, temperature gradation was introduced on the metallic culture vessel with the simple idea of using ice water and a hot plate. Consequently, a broad temperature gradation was immediately observed on the surfaces of the culture vessels. This simple but novel engineering method facilitated a comprehensive investigation into the thermal tolerance of cultured cells because cells can be exposed to several thermal stimulations. To demonstrate this argument, NHDF and MCF-7 cells were employed as model normal and cancer cell species, respectively; subsequently, the thermal tolerance of each cell species was evaluated. These results are qualitatively supported by those reported in previous research. As mentioned above, the proposed system provides a fundamental method for evaluating the thermal tolerance of cultured cells. Note that in this study, only the live/dead assay was considered to assess thermal tolerance because the main purpose of this study was developing a novel system. However, a thorough evaluation is required to accurately evaluate the thermal tolerance of cells. Detailed discussions supporting this argument are provided below, from the perspective of system development and cell responses reported in this study.

The proposed system uniquely employs a metallic material for the cell culture vessel, resulting in immediate temperature regulation and wide range of temperature gradations. The advantages of metallic vessels over conventional plastic vessels are shown in Figs. 3 and 4. Immediate temperature regulation was realized owing to the higher thermal conductivity of metallic materials compared to that of polystyrene. As shown in Fig. 4A and B, the temperature variation in the metallic materials was more rapid than that in polystyrene. In addition, a wide range of temperature gradations was observed owing to the high thermal conductivity. Polystyrene, which has a lower thermal conductivity than that of the metallic material, offers a relatively “thermally isolated” condition in the vessel, as indicated in Fig. 4C and D and Movies S1 and S2. Note that the thermal conductivities of pure titanium and polystyrene are approximately 17 and 0.10 to 0.14 W/mK, respectively. Furthermore, the hot plate, which could only be heated, was regulated to achieve a certain temperature by switching it on and off. Hence, the driving time of the hot plate with the plastic dish decreased, whereas the cold source (ice water) was constantly functional. Hence, the temperature distribution in the polystyrene vessel was shifted lower than that of the metal. Thus, the metallic culture vessel exhibited a wider temperature gradation. However, despite the advantages of employing a metallic vessel, its effects on cell functions during culturing must be examined. Accordingly, we fabricated metallic culture vessels with commercial-grade pure titanium via 3D printing. The material employed differed from that generally used for cell culturing. Although commercial-grade pure titanium is uncommon in culture vessel applications, previous studies have demonstrated that it is not cytotoxic [24]. Moreover, pure titanium exhibits corrosion resistance even in biological environments [25]. However, no study has reported the effects of the 3D printed pure titanium culture vessel surfaces treated with FPP on the functions of cultured cells. Furthermore, in addition to the challenges related to the new material, the geometric morphology of the culture surface affects cell activities. To focus on our main argument regarding the realization of a temperature gradation on the culture surface, we developed a culture surface with a homogeneous morphology using FPP, as shown in Fig. 2A to D. As shown in Fig. 2E to H, the cells were cultured without exhibiting cytotoxicity caused by the vessel. Although a different cell line was used in a previous study, Casp9, which is expressed in the early stage of apoptosis, was not upregulated owing to cell culture characteristics in pure titanium vessels [26]. Thus, it can be argued that employing a metallic culture vessel is effective for evaluating thermal tolerance. Furthermore, regarding the reuse of culture vessels, metallic materials are stable and durable; thus, even an autoclave can be used.

The results of this study are qualitatively reasonable considering existing research, which supports its validity. As shown in Fig. 5, NHDF cells have a stronger thermal tolerance compared to MCF-7 cells, which is consistent with existing results [18,27,28]. Furthermore, Fig. 7 shows that the thermal tolerance of NHDF was affected by cell density, whereas that of MCF-7 cells was not. This finding is consistent with the results published in a previous report [29], which supports the validity of our system. The area of dead cells after 24-h culturing was expanded. This is because apoptosis occurs for a longer time than necrosis, as previously reported [30]. Although qualitatively similar, quantitatively different results were obtained using this system. This can be attributed to the following 3 factors: First, in this study, the threshold temperature was measured based on the ratio of the areas of live and dead cells. While all live cells in this study were stained, only the nuclei of dead cells were stained. Thus, the threshold temperature did not directly indicate the lethal temperature. Second, the culture environment, such as the surface profile, may affect cell function [20,21]. In the field of mechanotransduction, mechanical stimuli such as shear stress, vibration, and other mechanical environments around cells have been studied as cues for regulating biochemical reactions [31–35]. Culture surface profile is a major research topic. It has been reported that the surface profile affects cell function, and the culture environment can affect the thermal response of cells [16,29,36,37]. Hence, this may be a factor that quantitatively generates different thermotolerances, which is an interesting research topic. Third, the concept of the realized temperature gradation in the vessel may be a reason. Cells secrete many hormones that regulate cell functions, which may regulate the functions of the parent cell as well as those of other cells; each effect is known as an autocrine and paracrine effect, respectively [38–40]. Cells exposed to different temperatures were cultured simultaneously in the same culture vessels implemented in this system, which has not been attempted in previous studies investigating thermal tolerance. Thus, this novel system with temperature gradation also realized a novel culturing environment from the perspective of chemical characteristics owing to the hormones secreted from the cells being exposed to different culture temperatures, which may be one of the factors causing the different results compared to those reported in previous research. Therefore, to eliminate the effect of chemical stimulation by hormones, a perfusable culture system [41] can be applied to proposed system in future studies. To use the developed system effectively, first the reaction of cells to thermal stimulation can be roughly estimated. Subsequently, thorough evaluations, such as western blotting or quantitative reverse transcription polymerase chain reaction, can be performed with another culture system in which a homogeneous culture temperature can be applied to the cells [18]. Cells cultured under the same temperature conditions can be collected from our culture system, provided that cells adhering to specific areas can be isolated. To achieve this, several techniques have been developed to collect specific cells [42–45]. Furthermore, although some cell lines were cultured in layers on the culture surface, the cell area was used as a measure of viability in this study [46,47]. This suggests that it is better to make a rough estimate with the developed system and use a different method to obtain an accurate evaluation. From the standpoint of obtaining more rigid data, the blurred borderline between live and dead cells depicted in Figs. 5 and 6 is an interesting approach. The variation in the thermal tolerance of cells owing to different cell densities on the culture surface may be one reason for this. Furthermore, in our system, cells could migrate during and after the thermal dosing, indicating that the thermally stimulated cells were exposed to various numbers of cells. Because various techniques to decrease the nonuniformity of seeded cell densities and speed of cell migration have been reported [48,49], such techniques can be applied to our system to obtain a more rigid borderline characterizing cell death. One factor that can blur the borderline is cell proliferation following thermal dosing, which can be halted by certain chemical substances. However, considering the natural proliferation rate, we also assessed the recovery level of the cells after thermal stress using the developed system. Furthermore, as mentioned above, although this study provides many research topics for future study, the results are reasonable considering those reported in previous research. Therefore, it can be argued that the present study developed a novel and convenient method to evaluate the thermotolerance of cultured cells. For clinical treatment, stimuli such as that provided by ultrasound, magnetic field, or laser, which regulate cell activity, are applied to raise the temperature of affected areas [50–54]. Multiple stimuli will synergistically regulate the activities of cells [55–57]. Thus, our device can be combined with other techniques to predict the clinical effects of hyperthermia using other stimuli.

Although it was demonstrated that the proposed system can be used for developing hyperthermia treatments, the system and idea of presenting a temperature gradation suggest novel directions for future studies. Furthermore, the proposed device is expected to be suitable for use in other research related to thermal stimulation of cells. In addition to the hyperthermia treatment of cancer cells, thermal stimulation is used for numerous other treatments, including physiotherapy [58–61]. Thus, such treatments should be improved using a database of cell responses to certain thermal stimuli, which can be offered by the proposed system. Furthermore, the proposed system can be used for the development of therapeutic treatments and fundamental research related to cell culture temperatures. This can aid in determining the proper cell culture temperature for each cell species, which is necessary for developing effective cell culture protocols. Culture temperature affects cell functions such as proliferation, migration, differentiation, and adhesion [23,62,63]. Since David Julius and Ardem Patapoutian received the Nobel Prize for their discoveries of receptors for temperature and touch, the relationship between temperature and cell functions has been attracting increasing attention [64–67]. Furthermore, mechanical environments such as the stiffness of the culture surface are known to regulate cell activity [68,69], which generates different cell functions in vitro than in vivo. To realize culture conditions similar to those in vivo by tuning the stiffness of the substrate around the cells, the culture surface of the developed culturing system can be covered with a gel or silicone rubber [68–70]. Even 3D cultures can be realized by preparing scaffolds on the culture surface. This may be useful for predicting the thermal tolerance of cells growing in vivo. Although numerous studies should be performed to realize an effective hyperthermia treatment, as mentioned above, the proposed system should have greater potential than the alternative development of hyperthermia for cancer therapy. Thus, it is considered that the proposed system offers fundamental knowledge that can be widely applied in the research field.

This study aimed to develop a culture system that can effectively measure the thermal tolerance of cells to develop a noninvasive cancer treatment. The key to realizing this effectiveness was the introduction of temperature gradation, which was realized by employing metallic culture vessels and a simple but unique temperature regulation system. Using the proposed culture system, the thermal tolerances of normal and cancer cells were evaluated; accordingly, reasonable results were obtained compared with those reported in previous studies. Furthermore, in addition to the original purpose of developing the culture system, the developed system can be used for other purposes to evaluate the thermal response of cells. Thus, the proposed system can be widely applied in future research.

## Data Availability

Data supporting the findings of this study are available in the article and supplementary information files or from the corresponding author upon request.

## Supplementary Materials

Supplementary 1 Note S1 Figs. S1 to S4 Tables S1 and S2 Movies S1 and S2
