# Supplementary material for: Titanium Culture Vessel Presenting Temperature Gradation for the Thermotolerance Estimation of Cells
Source: Cyborg Bionic Syst. 2023 Aug 7;4:0049. doi: 10.34133/cbsystems.0049 (PMC10405790; doi:10.34133/cbsystems.0049)
Supplement: Supplementary 1 — Note S1 Figs. S1 to S4 Tables S1 and S2 Movies S1 and S2 [file cbsystems.0049.f1.pdf]

Cyborg and Bionic Systems
A SCIENCE PARTNER JOURNAL

FRONT MATTER

Title

Titanium culture vessel presenting temperature gradation for the thermotolerance estimation of cells

Authors

Chikahiro Imashiro[1,2*], Yangyan Jin[3], Motoaki Hayama[3], Takahiro G. Yamada[4], Akira Funahashi[4], Katsuhisa Sakaguchi[5], Shinjiro Umezu[6], Jun Komotori[2*]

Affiliations

[1]School of Engineering, The University of Tokyo, Tokyo, 113-0033, Japan [2]Department of Mechanical Engineering, Keio University, Kanagawa 223-8522, Japan. [3]School of Integrated Design Engineering, Graduate School of Science and Technology, Keio University. [4]Department of Modern Mechanical Engineering, Waseda University, Tokyo 169-8555, Japan. [5]Department of Biosciences and Informatics, Keio University, Kanagawa 223-8522, Japan. [6]Department of Integrative Bioscience and Biomedical Engineering, Graduate School of Advanced Science and Engineering, Waseda University, TWIns, Tokyo 162-8480, Japan.
*Address correspondence to: email: cimashiro@g.ecc.u-tokyo.ac.jp, komotori@mech.keio.ac.jp

Supplementary Materials

Supplemental Note 1

Arithmetic mean roughness (*Sa*) is the arithmetic average of the absolute value of the height deviation from the mean surface. *Sa* is given by the equation

$$\boldsymbol{Sa = \frac{1}{A}\iint_A |Z(x,y)|dxdy}$$

where *Sa* is the arithmetic mean roughness of surface, and *A* is the surface area and *Z* is the height deviation from the mean surface. Maximum height roughness (Sz) is the vertical distance between the maximum peak height and the deepest valley depth within the measured area.

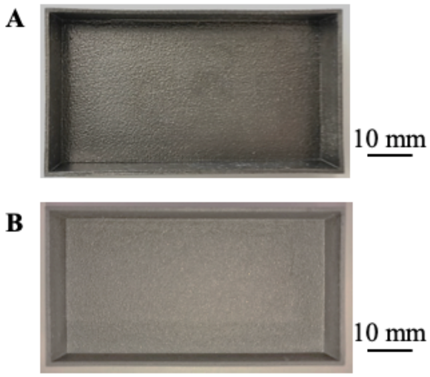

Fig. S1 Design and surface profile of the metallic culture surface. Upper view of the culture vessel before (A) and after (B) the FPP.

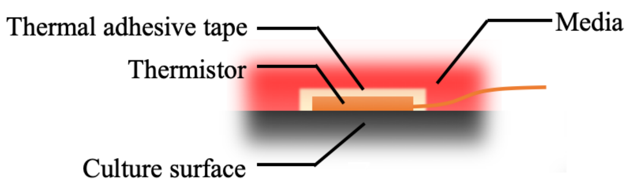

Figure S2: Schematic showing the thermistor adhered on the culture surface with a thermal adhesive tape. Further, for the stable measurement, the averaged temperature for 10 s was plotted every 10 s. Noting measurement was performed every 0.1 s.

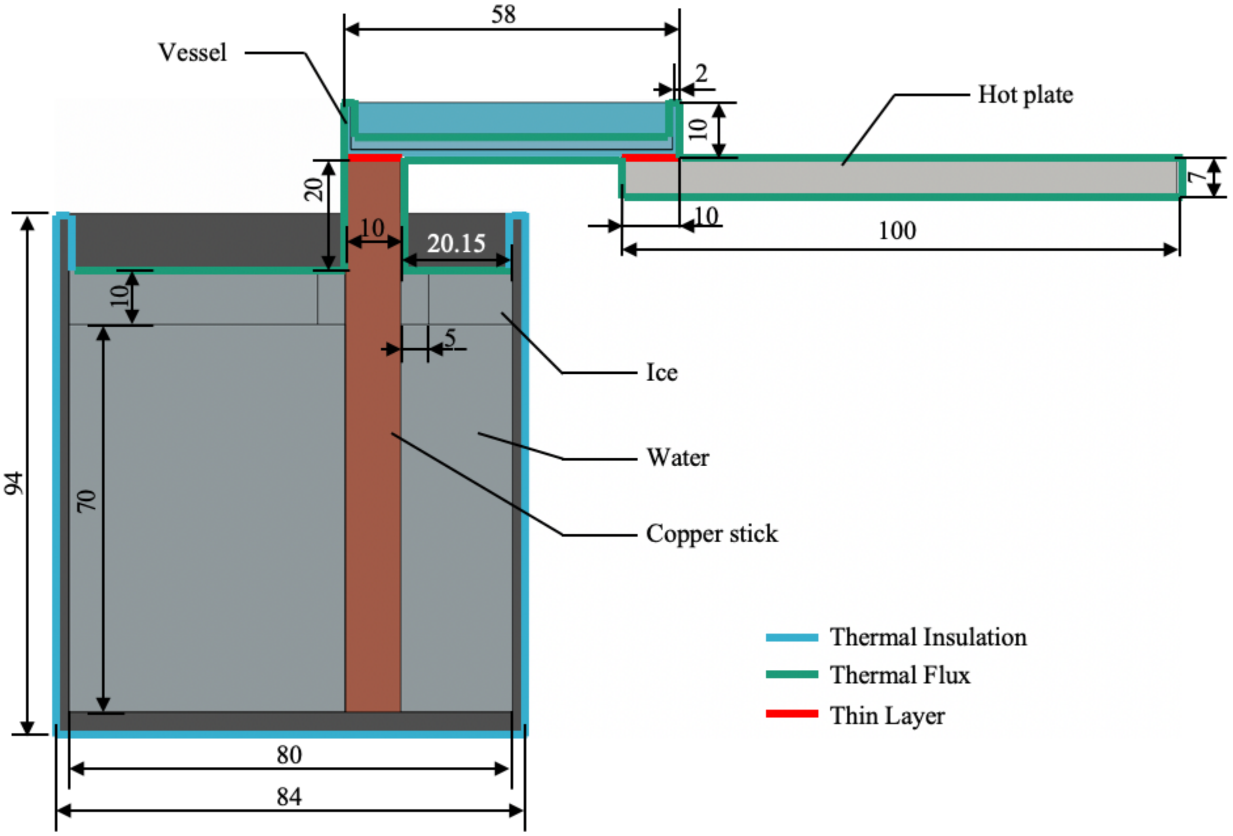

Figure S3: Side view of the simulation model. To simulate the device, temperature was regulated at two points shown in the figure; the temperature of point 1 was regulated at 0 °C, while that of point 2 was maintained within the range of 104 and 106 °C. To mimic the experiment the regulation of the point 2 temperature began when the temperature decreased to 104 °C, and heating was stopped when it reached 106 °C. Feature points were selected in the cell layer (15 µm above the culture surface) to measure the temperature of cells, and the cells were modeled as a thin layer with a thickness of 30 µm. The thermophysical property used for each material was obtained from COMSOL.

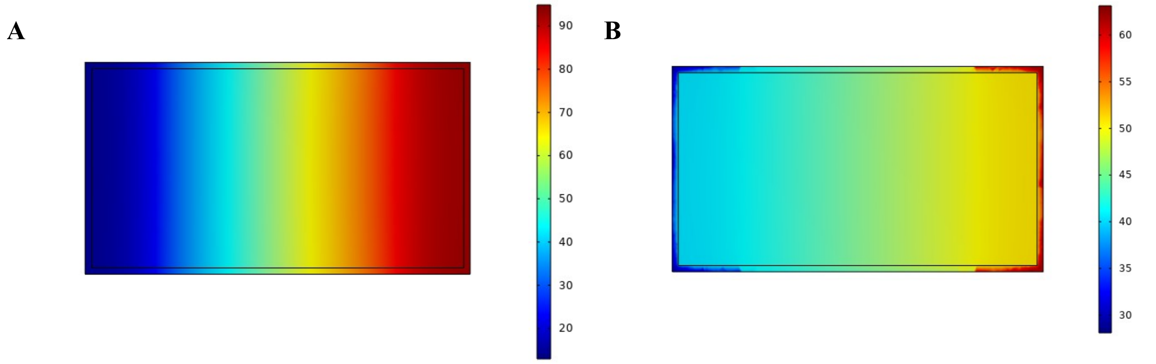

Figure S4: Comparison of the presented temperature distribution on metallic (A) and plastic (B) culture vessels using simulations. Upper images of the vessels made of metallic (A) and plastic (B) materials after 20 min of driving are shown.

Table S1 Results of the statistical analysis for Fig. 5B.

	Sum of square	Degree of freedom	F Value	P value
Interaction	1.159	1	0.1729	0.6885
Culture duration	34.17	1	5.095	0.0540
Cell species	38.63	1	5.759	0.0432
Residual	53.66	8		

Table S2 Results of the statistical analysis for Fig. 6B

	Sum of square	Degree of freedom	F Value	P value
Interaction	4.234	1	0.9382	0.3556
Culture duration	52.38	1	11.61	0.0067
Cell species	6.447	1	1.429	0.2596
Residual	45.13	10		
